# The Impact of Therapeutic Plasma Exchange on Inflammatory Markers and Acute Phase Reactants in Patients with Severe SARS-CoV-2 Infection

**DOI:** 10.3390/medicina59050867

**Published:** 2023-04-29

**Authors:** Tamara Mirela Porosnicu, Ioan Ovidiu Sirbu, Cristian Oancea, Dorel Sandesc, Felix Bratosin, Ovidiu Rosca, Daniel Jipa, Estera Boeriu, Satya Sai Sri Bandi, Marius Pricop

**Affiliations:** 1 Doctoral School, “Victor Babes” University of Medicine and Pharmacy Timisoara, Eftimie Murgu Square 2, 300041 Timisoara, Romania; 2 Intensive Care Unit, “Pius Brinzeu” Emergency Clinical County Hospital, 300723 Timisoara, Romania; 3 Center for Complex Network Sciences, “Victor Babes” University of Medicine and Pharmacy Timisoara, Eftimie Murgu Square 2, 300041 Timisoara, Romania; 4 Center for Research and Innovation in Precision Medicine of Respiratory Disease, “Victor Babes” University of Medicine and Pharmacy Timisoara, Eftimie Murgu Square 2, 300041 Timisoara, Romania; 5 Department of Anesthesia and Intensive Care, “Victor Babes” University of Medicine and Pharmacy Timisoara, Eftimie Murgu Square 2, 300041 Timisoara, Romania; 6 Department XIII, Discipline of Infectious Disease, “Victor Babes” University of Medicine and Pharmacy Timisoara, Eftimie Murgu Square 2, 300041 Timisoara, Romania; 7 Department of Pediatrics, Discipline of Pediatric Oncology and Hematology, “Victor Babes” University of Medicine and Pharmacy Timisoara, Eftimie Murgu Square 2, 300041 Timisoara, Romania; 8 Malla Reddy Institute of Medical Sciences, Suraram Main Road 138, Hyderabad 500055, India; 9 Discipline of Oral and Maxillo-Facial Surgery, Faculty of Dental Medicine, “Victor Babes” University of Medicine and Pharmacy Timisoara, Eftimie Murgu Square 2, 300041 Timisoara, Romania

**Keywords:** inflammatory markers, therapeutic plasma exchange, plasmapheresis, SARS-CoV-2, COVID-19

## Abstract

*Background and Objectives:* Due to the poor prognosis and the very high mortality rate associated with severe SARS-CoV-2 infections, various regimens have been tried to stop the evolution of the inflammatory cascade, such as immunomodulatory therapy and plasma clearance of the acute phase reactants involved. Therefore, the objective of this review was to analyze the effects of using therapeutic plasma exchange (TPE), also known as plasmapheresis, on the inflammatory markers of critically ill COVID-19 patients admitted to the intensive care unit (ICU). *Materials and Methods:* A thorough scientific database search was performed, and it included a review of articles published on PubMed, Cochrane Database, Scopus, and Web of Science from the beginning of the COVID-19 pandemic in March 2020 until September 2022 that focused on the treatment of SARS-CoV-2 infections using plasma exchange for patients admitted to the ICU. The current study included original articles, reviews, editorials, and short or special communications regarding the topic of interest. *Results:* A total of 13 articles were selected after satisfying the inclusion criterion of three or more patients enrolled with clinically severe COVID-19 that were eligible for TPE. From the included articles, it was observed that TPE was used as a last-resort salvage therapy that can be regarded as an alternative treatment method when the standard management for these patients fails. TPE significantly decreased the inflammatory status as measured by Interleukin-6 (IL-6), C-reactive protein (CRP), lymphocyte count, and D-dimers, as well as improving the clinical status measured with PaO_2_/FiO_2_ and duration of hospitalization. The pooled mortality risk reduction after TPE was 20%. *Conclusions:* There are sufficient studies and evidence to show that TPE reduces inflammatory mediators and improves coagulation function and the clinical/paraclinical status. Nevertheless, although it was shown that TPE decreases the severe inflammatory status without significant complications, the improvement of survival rate remains unclear.

## 1. Introduction

The new coronavirus SARS-CoV-2, unlike the other two viruses, MERS (Middle East respiratory syndrome) and SARS (severe acute respiratory syndrome), was a real challenge for the entire global health system due to the many cases that required hospitalization in a short period of time, causing a global crisis in the health system [1,2,3,4,5]. Regarding biological inflammatory markers, increased values of lactate dehydrogenase (LDH), ferritin, fibrinogen, interleukin-6 (IL-6), C reactive protein, D-dimers, and a reduced number of lymphocytes were described in multiple studies [6,7]. Many of the abnormalities identified in hospitalized cases were caused by a “cytokine storm”, characterized by an exaggerated host response to the virus with similar characteristics to bacterial septic shock and negative fulminant evolution that is usually fatal. In other words, the leading causes of death in patients with COVID-19 infection are ARDS and cytokine storm syndrome, which lead to multisystemic organ failure [8,9].

Depending on a population’s features and its associated risk factors, the mortality rate of severe COVID-19 cases continues to be significant. Despite the fact that several potentially useful therapeutic approaches have been investigated and tried, only a few of them have been shown to be successful, and only in particular circumstances [10,11]. For example, therapeutic plasma exchange (TPE), or plasmapheresis, is a medical procedure in which the patient’s plasma is removed from the morphotic components of their blood and then replaced with either an albumin solution or fresh frozen plasma (FFP) [12]. The primary objective of TPE is the removal of morbid components, such as pathogenic antibodies and inflammatory proteins [13,14].

Because a cytokine storm-mediated immune response is what causes organ damage, it stands to reason that removing damaging antibodies and cytokines might reduce the severity of the illness. The use of TPE in COVID-19 patients is based on the rationale that by removing the excess of proinflammatory cytokines, such as IL-6, tumor necrosis factor-alpha (TNF-α), and interleukin-1β (IL-1β), TPE can attenuate the cytokine storm and prevent the subsequent multiorgan failure and acute respiratory distress syndrome (ARDS) that are often observed in severe cases. The elimination of other fibrin breakdown products, such as D-dimers, might also contribute to an improvement in the hemostatic equilibrium [15,16]. In light of these considerations, TPE has lately been brought up as a potential supportive therapy option for severe SARS-CoV-2 infections. 

However, despite the growing interest in TPE as a potential therapy for severe COVID-19, the impact of TPE on the inflammatory markers and acute phase reactants in patients with SARS-CoV-2 infection remains inadequately studied. Therefore, the objective of this systematic review was to present the role of plasma exchange therapy in lowering inflammation markers and acute phase reactants in critically ill COVID-19 patients and finding the optimal treatment protocol to improve patients’ survival.

## 2. Materials and Methods

### 2.1. Study Design and Search Protocol

All relevant scientific papers discussing the use of TPE in severe SARS-CoV-2 infection were included in the analysis by using a structured and methodical search approach, which was carried out in accordance with the PRISMA criteria and PROSPERO guidelines [17]. The current systematic review was registered to the Open-Science Framework (OSF) platform. The search period was considered from the beginning of the COVID-19 pandemic in January 2020 until September 2022, focusing on the epidemiology, pathogenesis, diagnosis, COVID-19 treatment management, and immunopathology of the inflammatory markers in association with our topic of interest. 

Reference lists from the retrieved articles were manually examined for relevant information. PubMed, Cochrane Database, Scopus, and Web of Science were filtered using specific keywords, including {COVID-19}, {SARS-COV-2}, {severe inflammation}, {C-reactive protein}, {plasmapheresis}, {therapeutic plasma exchange}, {cytokine storm}, and {fibrinogen}. We used a combination of the following keywords: “therapeutic plasma exchange + COVID-19 + cytokine storm”; “plasmapheresis + COVID-19 + Cytokine storm”; “therapeutic plasma exchange + COVID-19 + critical care”; “therapeutic plasma exchange + COVID-19 + ICU”. 

In this systematic review, our primary objectives were to address two key questions related to the management of severe SARS-CoV-2 infection. First, we sought to determine the optimal treatment protocol for utilizing Therapeutic Plasma Exchange (TPE) in patients with severe COVID-19. Second, we aimed to identify the inflammatory markers that are most commonly influenced by the use of TPE in this patient population.

### 2.2. Inclusion and Exclusion Criteria

The inclusion criteria comprised the following: (1) original studies, reviews, editorials, case series with three or more patients, and short or special communications that focused on the management of severe SARS-CoV-2 infection; (2) hospitalized patients with SARS-CoV-2 infection older than 18 years; (3) having a positive polymerase chain reaction (PCR) for SARS-CoV-2 infection; (4) use of a controlled design for the administration of TPE.

The exclusion criteria comprised the following: (1) studies involving animal experiments; (2) publications that were not written in English; (3) case reports; (4) duplicate studies. The selected studies were evaluated by two different investigators independently, and complete texts were obtained only if both of them decided that the paper should be included. A third researcher was involved in case of divergent opinions. 

### 2.3. Data Extraction and Quality Assessment

For the purpose of our analysis, we retrieved the following information from the studies that were included: authors, year of publication, type of study, patient characteristics, concomitant therapies, time of TPE initiation and cessation, dose of TPE, type of replacement fluids, adverse effects associated with TPE, inflammatory markers, and outcomes.

**Figure 1:**
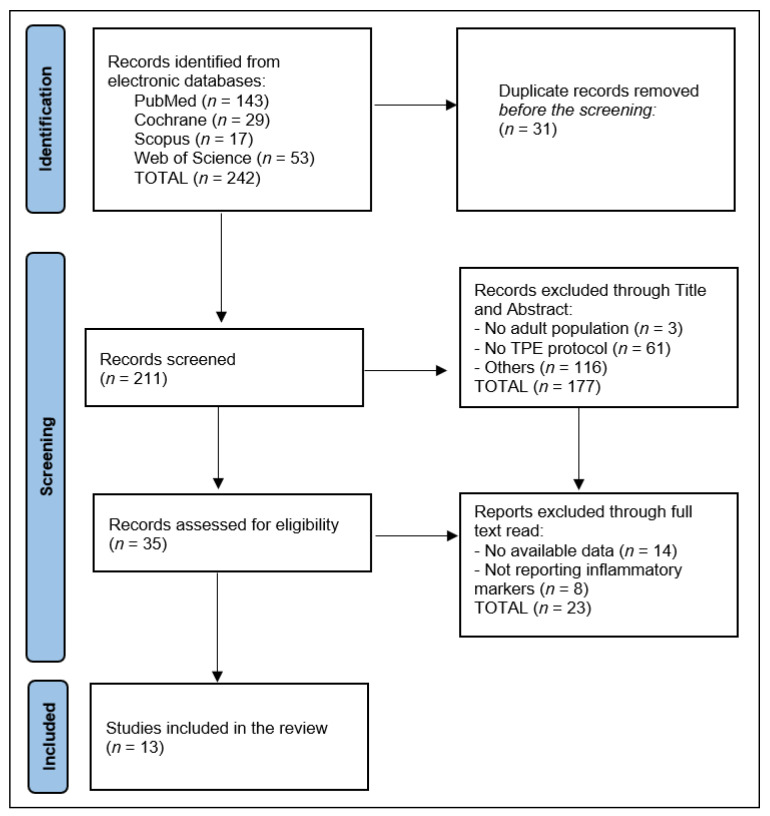
PRISMA flowchart.

All information was gathered from the articles’ text, tables, figures, and online supplemental resources. The selection procedure comprised the elimination of duplicate entries, abstract screening, and full-text screening. Initial results from the search returned 242 matching entries, of which 31 were duplicates. Figure 1 shows that 13 reports were included in the systematic review after abstract and title screening eliminated 177 studies, whereas full-text reading eliminated 23 studies.

Following the NHLBI-published Study Quality Assessment Tools, two researchers evaluated information from existing articles and reported results individually. The tools are unique to research designs and screen for any methodological or operational problems. The Quality Assessment Tool for Observational Cohort and Cross-Sectional Investigations was used for the remaining studies [18]. For each of the 14 questions for study evaluation, “Yes” replies were worth 1 point, whereas “No” and “Other” responses were worth 0 points. The final quality score was then calculated. Therefore, investigations with a rating from 0 to 4 were deemed to be of low quality, research with a grade between 5 and 9 was deemed to be of acceptable quality, and investigations with a score of 10 or more were deemed to be of good quality.

## 3. Results 

After filtering the matching studies, we included in this systematic review 13 articles that used TPE as treatment in adult patients with severe COVID-19 [19,20,21,22,23,24,25,26,27,28,29,30,31]. The included articles are summarized in Table 1. The main outcomes extracted from the studied papers observed a total of 485 severely ill patients treated with TPE, with a mortality rate of 18.1% (72 patients). However, the mortality rate is arguably lower than in patients without TPE treatment because only six studies had a control group for comparison [19,22,27,28,29,30,31], and their results were not always statistically significant. However, the duration of ICU admission was reported as significantly lower than in control groups, averaging six days compared with fourteen days among controls. 

TPE was usually performed multiple times, averaging three times during hospitalization and ranging from one to seven times. The main component of replacement fluid was fresh frozen plasma in all 13 studies, or FFP with a combination of citrate dextrose solution [19], human albumin and normal saline [21], normal saline only [22,29], or 5% human albumin only [23,24,25,26,27,30]. Regarding the safety of TPE, only one study reported one patient with associated hypotension [19], whereas another study reported femoral artery puncture in one patient and thrombophlebitis of the femoral vein in another patient [22]. 

Some key observations from the included studies comprise reduced inflammatory markers [19,21,22,24], reductions in SOFA scores [19,23], and the conclusion of better results in critically ill patients when TPA is used early after disease onset [19,22,26,29]. Another important finding is improved oxygenation after plasmapheresis, which was reported in five of the analyzed studies [20,21,23,25,28].

**Figure 2:**
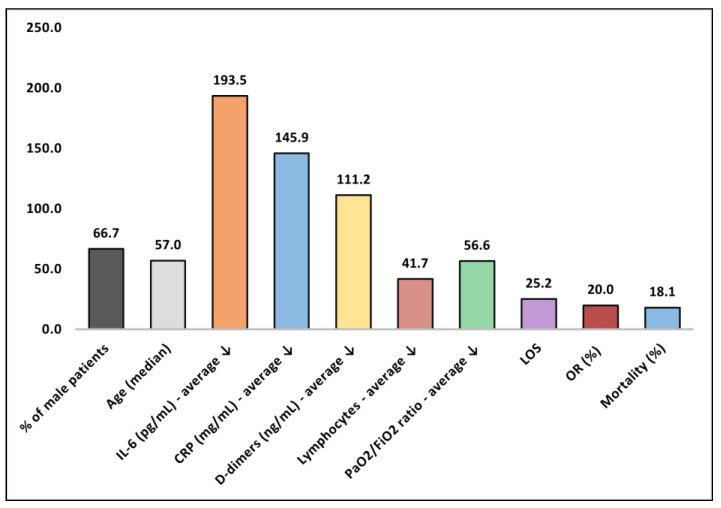
Summary of findings representing the average values of variables reported in the included studies, and the average decrease (down arrow) of inflammatory markers after TPE procedure; IL-6—Interleukin-6; CRP—C-reactive protein; PaO_2_—Partial pressure of oxygen; FiO_2_—Fraction of inspired oxygen; LOS—length of stay in hospital (days); OS—Overall survival.

Regarding the change in inflammatory markers observed in patients with severe COVID-19, it was observed in all studies that reported a change in variation that inflammatory markers significantly decreased after undergoing TPE. The highest reduction of inflammatory markers was observed in a study by Khamis [19], which reported a 336 mg/mL decrease of CRP. A study by Zaid [26] reported a 574 pg/mL decrease in IL-6 levels post-TPE. However, data is inconsistent regarding the timing of serum marker measurement, or how long after TPE the measurements were taken. As seen in Table 2 and Figure 2, most of the patients were men (66.7%) who had an age range between 48–75 years and a median age of 57 years. The average IL-6 reduction after TPE was 193.5 pg/mL; CRP decreased by an average of 145.9 mg/L, whereas D-dimers decreased by 111.2 ng/mL. Lymphocyte count was also significantly lower after TPE with a 41.7 × 10^9^/L decrease. The length of hospitalization, however, was very long; studies reported an average of 25.2 days of admission. The mortality rate varied significantly, which is probably due to the severity of patients involved, from 0.0% in some of the studies [24,25,26,28] up to 60.0% in the study described by Matsushita et al. [20] and an overall average of 18.1%. Lastly, only four studies reported the odds ratio for improvement after TPE compared with a control group [21,27,29,30,31], but data showed a significant improvement in clinical and inflammatory status of the patients from 17% to 32%.

## 4. Discussion

### 4.1. Supporting Literature for the Safety and Efficacy of TPE 

In our study, we investigated the impact of TPE on various inflammatory markers, including CRP, IL-6, ferritin, and lymphocyte counts. The significant reduction in these inflammatory markers after TPE suggests that this treatment effectively mitigates the cytokine storm, which is a major contributing factor to the severe manifestations of COVID-19. The cytokine storm is characterized by an excessive and uncontrolled release of pro-inflammatory cytokines, leading to acute respiratory distress syndrome (ARDS), multi-organ failure, and ultimately, death. The TPE treatment’s ability to suppress this excessive inflammatory response may explain the observed improvement in clinical outcomes, such as oxygenation and SOFA scores, among the treated patients [32,33,34].

Fahad Faqihi et al. noted that patients receiving TPE showed marked and sustained increases in lymphocyte count and significant decreases in CRP, LDH, ferritin, D-dimers, and IL-6. This study demonstrates that TPE reduces inflammatory markers and improves oxygenation and the clinical status of patients with life-threatening forms of COVID-19. However, it does not significantly affect mortality at 35 days [27]. The observed increase in lymphocyte counts following TPE treatment is particularly notable in the context of COVID-19, as lymphopenia has been identified as a common laboratory abnormality in patients with severe disease. Lymphocytes play a crucial role in the immune response to viral infections, and their depletion in COVID-19 patients is associated with a higher risk of severe outcomes. By increasing lymphocyte counts, TPE may contribute to enhancing the immune response against SARS-CoV-2 and facilitating recovery.

Similarly, Khamis et al. observed a significant decrease in inflammatory markers and an increase in lymphocyte count after TPE. As a result, patients receiving TPE had an improved clinical course compared to patients in the control group in terms of extubation and mortality rates. Patients receiving TPE were also less likely to develop severe pneumonia by comparison to those in the control group [19]. In addition, a case series study by Zaid et al. proved the effectiveness of plasma exchange therapy by lowering pro-inflammatory cytokines and ameliorating the cytokine storm [26], as all patients had increased inflammatory markers before TPE and presented good clinical and biological courses afterward. According to the authors, the improved evolution was due to prompt TPE initiation (on the first day of the “cytokine storm”) before intubation, and mechanical ventilation was needed.

Molecules with a molecular weight of less than 1000 kDa, such as interleukins, can be filtered through TPE. All of the inflammatory markers and acute phase reactants mentioned in this paper have a molecular weight low enough to be effectively eliminated through TPE, decreasing the inflammatory burden and suppressing cytokine release syndrome. Lu et al. observed much higher serum values of IL-1beta in the blood of COVID-19 patients compared to non-COVID patients, but they also noted higher cytokine values in general in patients admitted to the ICU [34].

According to other studies, IL-1 plays a key role in inducing the cytokine storm that appears in severe COVID-19 cases [23]. This cytokine storm can lead to acute lung injury (ALI), systemic inflammatory response syndrome (SIRS), or ARDS. IL-1 also promotes bronchial and alveolar inflammatory responses in patients with pulmonary tissue damage. In addition, it can stimulate hepatocytes to produce acute phase reactants. Several studies have shown that IL-1 controls the biosynthesis of IL-6, which is known to be one of the major negative prognostic factors in COVID-19 [35]. Thus, uncontrolled production of IL-1beta may be an underlying factor in acute lung injury and cytokine storm in SARS-CoV-2-infected patients. In view of this, the inhibition or purging of IL-1 may be extremely beneficial in the treatment of cytokine storm syndromes.

Even from the beginning of the new coronavirus pandemic, IL-6 has been a prognostic biomarker with important clinical value that has distinguished between mild, moderate, and severe forms of the disease, raising the important question of whether controlling IL-6 could prevent the severity of SARS-CoV-2 infection. In many studies, attempts were made to control IL-6 by keeping it within normal limits through various medical procedures. One of the procedures was using therapeutic plasma exchange, which significantly reduced IL-6 levels [24,36,37,38,39].

Recent literature data show that IL-6 is one of the biomarkers involved in severe inflammatory syndrome, but a more detailed analysis shows that its value is lower in bacterial septic shock [40]. In severe inflammatory syndrome from coronavirus type 2 infection, the clear impact of interleukin-6 on mortality and morbidity has been demonstrated; basically, the multiple system organ failure in the clinical picture is caused by severe inflammatory syndrome and not by direct action of the virus. In a retrospective longitudinal cohort study conducted by Manson et al., an increase in IL-6 was associated with an increase in oxygen demand and an increase in noninvasive and invasive mechanical ventilation support, resulting in increased mortality [38]. In a study conducted in the USA on 289 patients, Ashrafzadeh-Kian et al. showed the usefulness of IL-6, being the most reliable biomarker as a disease severity predictor in COVID-19, as it was correlated with the hospitalization duration, disease severity, and prognosis. The author pointed out that the test can be used in the triage of infected patients [39].

In the context of SARS-CoV-2 infection, elevated CRP values have been associated with tissue destruction, poor prognosis, and, implicitly, increased mortality [40]. Elevated CRP levels in the early stages of COVID-19 have been associated with lung destruction and disease severity. According to lung investigations using computed tomography corroborated with the laboratory analyses, the increase in CRP level was found before the appearance of lung lesions; thus, it was considered that CRP has a predictive value on disease severity [41].

According to a study conducted in China by Ling et al., there is a strong correlation between the CRP and albumin ratio, sequential organ failure assessment (SOFA) score, and the length of hospitalization in surviving COVID-19 patients. In the acute inflammatory stage, there is an increase in CRP and a decrease in albumin values. These changes are not only an indicator of the disease severity but even mortality risk factors in patients with severe COVID-19, as they indicate the cytokine storm’s debut [42,43]. Another study conducted in Spain suggests that a CRP value above the threshold of 9.1 mg/dl and a SOFA score higher than 2 in COVID-19 patients at the time of admission are independent predictors (with a sensitivity and specificity of 77%) of admission to the ICU [44].

Studies show that plasma exchange is more likely to alter pathogenic immunological drivers than antibody-mediated immune responses in severely ill COVID-19 patients. This might lower D-dimer levels depending on their molecular weight, suggesting a contrived reduction rather than a genuine recovery in the patient’s illness. Moreover, the findings presented are consistent with those of other studies, in which the authors demonstrated a beneficial effect of plasma exchange by demonstrating decreased fatality rates in patients with D-dimers greater than or equal to 2 mg/L on plasma exchange therapy compared to patients with D-dimers >2 mg/L without plasma exchange [45]. Despite the severity of the illness, these findings additionally reinforce the hypothesis that TPE improves survival by demonstrating a 30-day death rate of 32.1% in TPE-treated patients compared to 57.1% in patients receiving conventional therapy.

Fibrinogen is a large molecule with a molecular weight of approximately 340 kDa that plays an important role in blood clotting, inflammatory response, cellular interactions, wound healing, and neoplasia. It is considered an acute phase reactant present in many clinical syndromes with procoagulant status, such as severe bacterial infections, various neoplasms, and almost all moderate and severe forms of COVID-19. A study by Kuluöztürk M. et al. described the correlation between fibrinogen and albumin ratio, lung damage, and C-reactive protein values, concluding that a fibrinogen/albumin ratio of over 144.59 may be an early prognostic marker and may predict admission to ICU [46].

One of the major challenges in the therapeutic management of COVID-19 patients (especially in the ICU) is the clogging of filters, plasma filters, cytokine filters, and ECMO systems. This has been attributed to a procoagulant status found in patients with SARS-CoV2 infection. Compared with the literature, Zarbock et al. showed the importance of using citrate anticoagulation during dialysis sessions, concluding that filters have a longer lifespan if citrate is used rather than heparin [47]. In another study conducted last year by Sui et al., which included 119 COVID-19 patients with high fibrinogen values, fibrinogen is shown to be closely correlated with several biological abnormalities such as elevated inflammatory markers, multiple organ dysfunction, intensive care admission, and higher mortality [48].

Erythrocyte sedimentation rate (ESR) was recognized as an acute phase reactant used to indicate systemic inflammation. Although the increase in ESR in COVID-19 patients cannot be fully explained, it is suspected to be due to changes in erythrocyte forms and plasmatic changes [49]. Mahat et al. performed a meta-analysis on the dynamics of the inflammatory markers in COVID-19, which included 83 patients. It was observed that the severe group of patients had higher ESR values compared to the mild/moderate group, thereby being associated with the severity of the disease [50]. This increase in ESR in severe cases of COVID-19 reflects a profound inflammatory response and a strong expression of acute phase proteins.

An interesting link between ESR and coagulation is described by Al-Samkari et al. in a study on 400 hospitalized COVID-19 patients. The authors showed that patients with thrombotic complications or bleeding had higher ESR values than patients without thrombotic complications or bleeding. It was concluded that elevated ESR values at admission are predictive of thrombotic complications during hospitalization [51]. In addition, elevated ESR values at admission were predictive of both severe disease and mortality. The authors concluded that high values of ESR and other inflammatory markers correlate with the disease severity and the risk of death.

LDH is considered an inflammatory marker that indicates acute or chronic tissue destruction. An increase in serum LDH has also been reported in acute lung injury caused by interstitial lung disease and severe respiratory failure [52,53]. In addition, it is considered to be one of the strongest biomarkers associated with mortality in ARDS. LDH is independently associated with one-month mortality in elderly patients with COVID-19 and is also a respiratory failure predictor in hospitalized, SARS-CoV-2-infected patients [54,55]. In a pooled analysis by Henry et al. containing nine studies with 1532 patients, it was found that elevated LDH values were associated with a sixfold increased risk of developing severe COVID-19 and a 16-fold increase in mortality rate [56]. According to Masumoto et al., serum LDH values > 355U/L at admission were associated with an increased risk of death in patients with COVID-19 and cardiac comorbidities [57]. 

Elevated LDH levels may reflect the destruction of pneumocytes, myocardial cells, and other organs. According to a study by Hachim et al. on 541 patients, it was observed that patients with severe or critical forms of COVID-19 had much higher LDH values compared to patients with mild or moderate forms, and patients admitted to the ICU had higher LDH values compared to those treated in non-critical wards; non-survivors also had elevated serum LDH values compared to survivors, and higher LDH values were found in patients with ARDS compared to patients without ARDS [58]. Zheng et al. conducted a retrospective study comparing computerized tomography scans and biological investigations of 231 COVID-19 patients. The results suggest that the severity of the lung damage found on CT scans correlates positively with age and plasma values of ESR, D-dimers, LDH, and CRP [59]. Similarly, a study involving approximately 400 COVID-19 patients observed that elevated LDH values were found in deceased patients compared to survivors (median 702 vs. 498 U/L, *p* < 0.001), concluding that LDH values higher than 400 IU/L are associated with increased mortality in COVID-19 patients [60].

During the COVID-19 pandemic, abnormally elevated levels of ferritin were observed in patients with SARS-CoV-2 infections, bringing into question the correlation between the value of ferritin and their prognosis. The common connection between hyperferritinemia syndrome and the aforementioned complications is the combination of elevated ferritin levels and life-threatening inflammation, which will eventually lead to multiple organ failures. Although the exact link between COVID-19 and ferritin is not known at the cellular level, the literature shows many links between the severity of the disease and ferritin [61]. Ferritin levels were higher in non-surviving patients than in survivors as well as in those admitted to the intensive care units (ICU) who needed mechanical ventilation as opposed to those treated in other medical stations and those who did not require mechanical ventilation [62]. 

In a study by Carubbi et al., ferritin percentiles were calculated, and it was concluded that patients with ferritin values above the 25th percentile were more common in men and had higher fibrinogen, LDH, and procalcitonin levels in comparison with those who had ferritin values below the 25th percentile [63]. It should also be noted that lung damage, prevalence of septal thickening, and enlargement of the mediastinal lymph nodes were more pronounced in patients with elevated ferritin values, and functional impairment (PaO2/FiO2) was also more severe. Ferritin levels above the 25th percentile were also associated with the involvement of all five lung lobes, the presence of septal thickening, and the presence of mediastinal lymphadenopathy, regardless of age and sex [64]. In Pakistan, 78% of COVID-19 patients included in a study had elevated ferritin values, showing that the mean values of ferritin, D-dimers, CRP, and LDH were higher in patients with severe symptoms than in patients with mild or moderate symptoms. Patients with mild symptoms showed a mean value of 732.73 ng/mL, patients with moderate symptoms, 801.96 ng/mL, and patients with severe symptoms, 819.85 ng/mL [64].

### 4.2. Study Limitations

A limitation of our paper is that only the usual inflammatory markers involved in the cytokine storm were analyzed, limiting the complete evaluation of the inflammation profile. Five of the included articles were case series, which often provide positive results; hence, there is a substantial publication bias, and the aforementioned investigations should be evaluated with care. However, the total number of patients that underwent TPE was 485, which contributes to the overall significance of the observed findings. In addition, the COVID-19 diagnosis and treatment protocol were not standardized throughout all studies. The included studies are not homogenous regarding clinical indications, timing of TPE initiation, number of sessions, time intervals between sessions, and type of replacement fluid differed amongst trials; thus, additional data regarding these factors would be necessary for future research. Therefore, the studies included in the present systematic review did not allow us to adequately provide an optimum TPE therapy regimen.

## 5. Conclusions

TPE could be regarded as an alternative therapy complementary to standard treatment in severely ill COVID-19 patients. Most studies describe a reduction in inflammatory mediators and improvement of coagulation function, as well as clinical status, compared with admission features after three to five TPE sessions. However, the described studies are not standardized, and the results show inconsistency between them; the majority report no significant improvement after TPE in comparison with a control group. In addition, some studies suggest that TPE improves survival rate by correcting the inflammatory status of the patients without significant side effects, although it is not yet statistically proven in trials with a higher number of patients to offer reliability. Thus, it is important to emphasize the need for more well-designed randomized controlled trials with larger sample sizes and standardized protocols to determine the true efficacy, safety, and optimal treatment protocol for TPE in the management of critically ill COVID-19 patients. This will help provide more robust and reliable evidence for the clinical application of TPE in this patient population.

## Figures and Tables

**Table 1:** Literature review of researched articles.

First Author, Country	Type of Study	Number of Patients	Number of TPE Treatment	Replacement Fluid	TPE Safety	Special Observations/Conclusions
F. Kharmis, Oman [19]	Case–control series	11 TPE 20 Control group	5	FFP, citrate dextrose solution	Hypotension (1 patient)	Reduced inflammatory markers and SOFA scores. TPE should be utilized earlier in critically ill patients within 7–14 days of illness onset.
Y. Matsushita, Japan [20]	Retrospective study	5	3–7	FFP	Not reported	Positive evolution in 40% of patients. Decrease in CRP and improvement in PaO2/FiO_2_ ratio in all cases.
S. M. Hashemian, Iran [21]	Single group case series study	15	3	5% human albumin solution and 0.9% saline. FFP from with positive detection anti-SARS COV-2 IgG and IgM (4 patients).	Not reported	Improvement in oxygenation status. Reduced inflammatory mediators *p* < 0.001. Improvement in hepatic functions. TPE offers safety and efficacy in removing inflammatory cytokine and acute phase proteins.
S. M. Kamran, Pakistan [22]	Retrospective observational study	45 TPE 45 control group	1–5	FFP and normal saline 2:1	Femoral artery puncture (1 patient) Thrombophlebitis of the femoral vein (1 patient)	Decreased duration of hospitalization. Reduced inflammatory markers. Better results of TPE when used closer (within 12 days) to onset of symptoms.
F. Faqihi, Saudi Arabia [23]	Prospective study	10	5–7	FFP or human albumin 5%	None	Significantly reduced inflammatory markers and improved PaO2/FiO_2_ ratios and SOFA scores.
J. Fernandez, Spain [24]	Single center case series study	4	2–6	Human albumin 5%	None	Reduced inflammatory markers. Effective rescue therapy in critically ill patients. Improved survival in very severe COVID-19 therapy. Decreased in severity scores.
W. L. Gluck, USA [25]	Single center case series study	10	4–5	FFP or human albumin 5%	None	Reduction in inflammatory markers. Improved oxygenation parameters. 4/4 of patients were liberated from supplemental oxygen. 2/6 patients were extubated within 14 days.
I. Zaid, Marocco [26]	Retrospective case series study	7	3–5	FFP	None	Significant reduction in inflammatory markers. TPE should be used earlier in critically ill patients.
F. Faqihi, Saudi Arabia [27]	Randomized controlled clinical trial study	43 TPE 44 control group	1–5	FFP or human albumin 5%	None	Decrease in inflammatory markers. Increased lymphocytes and ADAMTS-13 activity. Duration of hospitalization in ICU was reduced in the TPE group. Faster clinical recovery decreased the SOFA score for TPE patients.
M. Hassaniazad, Iran [28]	Retrospective clinical study	22 TPE 22 control group	3	Human albumin 5%, normal saline, FFP	None	TPE can effectively improve clinical symptoms and reduce inflammatory markers.
Z. Jamil, Saudi Arabia [29]	Retrospective cohort study	81 TPE 81 control group	5	FFP, normal saline	None	Reduction of inflammatory markers. Improved PaO2/FiO_2_ ratio. Days of mechanical ventilation were reduced compared with the control group. Higher rate of survival in TPE group.
L. Cegolan, Iran [30]	A retrospective observational controlled study	43 TPE 30 control group	1–5	50% FFP + 50% human albumin 5%	None	Reduction of inflammatory markers. Mortality was lower in the TPE group due to the lower severity of patients with COVID-19.
CJ. Diskin, USA [31]	Prospective observational	42 TPE 147 controls	5	FFP, convalescent plasma	2 patients with “minor reactions”	Reduction of inflammatory markers. Higher rate of survival in TPE group. PaO_2_/FiO_2_ ratio in all cases.

TPE—Therapeutic Plasma Exchange; FFP—Fresh Frozen Plasma; SOFA—Sequential Organ Failure Assessment; ICU—Intensive Care Unit; PaO2/FiO2 ratio—The ratio of arterial oxygen partial pressure (PaO2 in mmHg) to fractional inspired oxygen; CRP—C-reactive protein.

**Table 2:** Patients’ outcomes identified in the studied articles.

No.	Quality Assessment	Male%	Age *	IL-6 (pg/mL)	CRP (mg/L)	D-dimer (ng/mL)	Ly (×10^9^/L)	PaO2/ FiO2	LOS	OR%	Mortality
1 [19]	Acceptable	100	50	334	336	23	60	15	19.0	NR	9.1%
2 [20]	Low	80.0	75	NR	NR	NR	NR	NR	31.6	NR	60.0%
3 [21]	Acceptable	60.0	57	26	188	NR	NR	40	9.6 (ICU)	17%	40.0%
4 [22]	Low	92.0	60	17	250	150	54	NR	15.0	NR	17.9%
5 [23]	Good	70.0	51	128	58	65	55	23	15.0 (ICU)	NR	10.0%
6 [24]	Low	100	57	20	66	81	NR	40	41.2	NR	0.0%
7 [25]	Low	30.0	52	26	123	NR	NR	43	NR	NR	0.0%
8 [26]	Low	57.1	57	574	133	NR	42	20	20.2	NR	0.0%
9 [27]	Good	82.8	48	423	201	40	50	165	19.0 (ICU)	19%	20.9%
10 [28]	Acceptable	50.0	61	NR	180	N	21	76	NR	NR	0.0%
11 [29]	Good	24.7	56	NR	24	308	10	69	NR	19%	19.8%
12 [30]	Acceptable	50.0	NR	NR	146	NR	NR	22	NR	32%	14%
13 [31]	Good	70.7%	60	NR	46	NR	NR	110	24.1	13%	43.9%

* Data reported as medians, unless specified differently; IL-6—interleukin-6; LOS—length of stay in hospital (days); Ly—Lymphocytes (×10^9^/L); OR—Odds Ratio for exposure to TPE (% of increased survival after TPE). Laboratory markers reported as the difference between pre-treatment and post-treatment with TPE; PaO2/FiO2 ratio—The ratio of arterial oxygen partial pressure (PaO2 in mmHg) to fractional inspired oxygen; NR—Not reported; ICU—Intensive care unit; CRP—C-reactive protein.

## Data Availability

Not applicable.
